# Supplementary material for: New Insights into Microbial Degradation of Cyanobacterial Organic Matter Using a Fractionation Procedure
Source: Int J Environ Res Public Health. 2022 Jun 7;19(12):6981. doi: 10.3390/ijerph19126981 (PMC9222324; doi:10.3390/ijerph19126981)
Supplement: Supplementary file 1 [file ijerph-19-06981-s001.zip › ijerph-1738594-supplementary.pdf]

International Journal of
Environmental Research and Public Health

MDPI

Supplementary Materials

Text S1

Main Components of Fluorescent Dissolved Organic Matter (FDOM)

The main components of FDOM were analyzed by fluorescence spectroscopy technique coupled with parallel factor analysis [47]. A fluorescence spectrophotometer (Hitachi F-4000) with a scanning speed of 2400 nm/min was employed to measure the excitation-emission matrice (EEM). The EEM fluorescence spectra was gathered with scanning emission (Em) wavelength from 250 to 600 nm at 1 nm increments, and the excitation (Ex) wavelength from 200 to 450 nm at 5 nm increments, respectively. The blank scans were performed every 10 analyses using Milli-Q water. And Rayleigh and Raman scattering (peak emission ±10–15 nm at each excitation wavelength) were removed from the EEM spectra, and then filled in the missing regions by the three-dimensional Delaunay interpolation of the surrounding data points [70]. The chromophoric DOM (CDOM) absorbance was used to correct the measured EEMs to eliminate the inner-filter effect, as described in previous studies [71,72]. During the experiments, a total of 150 EEMs spectra were obtained for PARAFAC analysis. The MATLAB (Mathworks, Natick, MA, USA) together with the DOM Fluor toolbox (http://www.models.life.ku.dk/ accessed on 6 May 2022) were employed for data analysis [48,49].

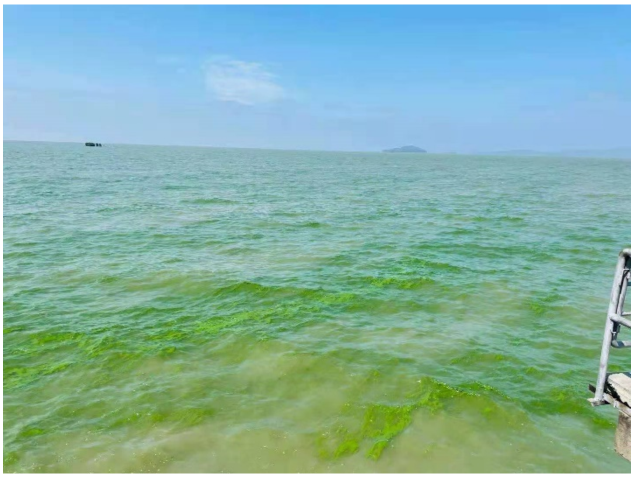

Figure S1. The cyanobacterial blooms in China were recorded during the summer of 2021 off the lakeside of Taihu. Photo shows the *Microcystis* dominated total phytoplankton community growing in the near lake waters and deposited on the beaches of the countryside.
